# The diketopiperazine-fused tetrahydro-β-carboline scaffold as a model peptidomimetic with an unusual α-turn secondary structure

**DOI:** 10.3762/bjoc.9.17

**Published:** 2013-01-22

**Authors:** Francesco Airaghi, Andrea Fiorati, Giordano Lesma, Manuele Musolino, Alessandro Sacchetti, Alessandra Silvani

**Affiliations:** 1 Dipartimento di Chimica, Università degli Studi di Milano, via Golgi 19, 20133 Milano, Italy; 2 Dipartimento di Chimica, Materiali ed Ingegneria Chimica ‘Giulio Natta’, Politecnico di Milano, piazza Leonardo da Vinci 32, 20132 Milano, Italy

**Keywords:** α-turn, conformational analysis, diketopiperazine, peptidomimetics, tetrahydro-β-carboline

## Abstract

Aiming at restricting the conformational freedom of tryptophan-containing peptide ligands, we designed a THBC (tetrahydro-β-carboline)-DKP (diketopiperazine)-based peptidomimetic scaffold capable of arranging in an unusual α-turn conformation. The synthesis is based on a diastereoselective Pictet–Spengler condensation to give the THBC core, followed by an intramolecular lactamization to complete the tetracyclic THBC-DKP fused ring system. The presence of conformers bearing the intramolecular thirteen-membered hydrogen bond that characterizes the α-turn structure is confirmed by ^1^H NMR conformational studies. To the best of our knowledge, this scaffold represents one of the rare examples of a designed constrained α-turn mimic.

## Introduction

**Figure 1:**
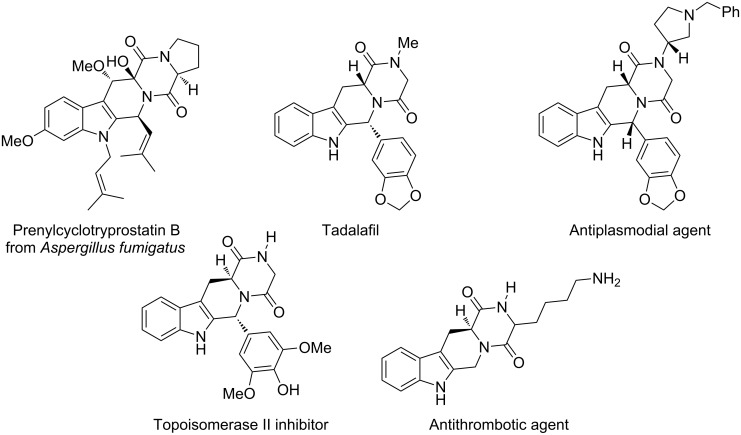
THBC-DKP-based natural and synthetically made compounds.

From a long time, the alkaloids containing the 1,2,3,4-tetrahydro-β-carboline (THBC) skeleton have represented important lead structures in view of their wide range of biological activities [1], mainly due to their interaction with the central nervous system [2–7]. Moreover, recently some tetracyclic β-carbolines have been described to act as selective inhibitors in the anticancer field [8–9], or to be endowed with antimalarial properties [10]. 2,5-Diketopiperazine (DKP)-based compounds are heterocyclic scaffolds structurally similar to peptides. They have attracted attention in recent years because of their broad biological activities [11–12] and therapeutic applications, ranging from antibiotics [13] to anticancer agents [14]. Moreover, the DKP moiety has been exploited as a peptidomimetic scaffold [15–17]. Structural unification of THBC and DKP pharmacophores has led to new classes of biologically active tetracyclic compounds, both naturally occurring and synthetically made [18]. Highly complex natural products displaying the fused, tetracyclic THBC-DKP ring system were recently isolated from the fungus *Aspergillus fumigatus* [19] and have been shown to exhibit significant cell growth inhibitory activities against various cell lines [20]. Specifically designed THBC-DKP-based compounds have received considerable attention over the past few years for their valuable biological activities, ranging from the inhibition of the cyclic guanosine monophosphate type 5 specific phosphodiesterase (PDE 5) for the treatment of erectile dysfunction (Tadalafil) [21], and the inhibition of plasmodial PDE activity for antimalarial drugs [22–23], to topoisomerase II inhibition [24], and oral antithrombotic properties [25] (Figure 1).

In our ongoing program of design of pharmacophore-based combinatorial libraries [26] and identification of new peptidomimetic scaffolds of potential interest in drug discovery [27–30], we evaluated the THBC-DKP-based scaffold as a potential suitable motif for the creation of unusual reverse-turn nucleators.

Reverse turns are structural motifs commonly found in bioactive peptides, which, besides being fundamental in protein folding, play a central role as molecular-recognition elements [31–34]. In addition to the most frequently occurring β and γ-turns, reversal of the polypeptide chain direction in globular proteins can also occur thanks to less common substructures, for example involving five amino acids residues, such as the case of the α-turns.

Even if a very large majority of α-turn segments form a part of regular α-helices, isolated α-turns have been reported [35], which are stabilized by a 5→1 hydrogen bond between the carbonyl oxygen at position *i* and the amide at position *i*+4.

Quite recently the presence of α-turns in constrained peptides has been associated with various relevant biological activities, highlighting potential applications in the field of bacteriolytic [36], antiviral [37] and anti-HIV compounds [38]. Other examples of α-turn conformations are described in synthetic peptidomimetics [36,39–42].

**Figure 2:**
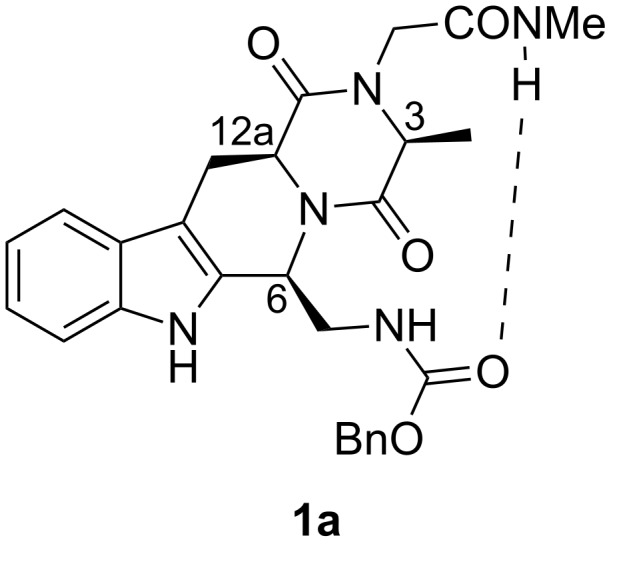
THBC-DKP-based peptidomimetic **1a**.

Despite the growing interest in this kind of reverse turn and the need for all kinds of conformationally constrained mimics as tools for medicinal chemistry, the development of constrained α-turn mimetics has received little attention until now. We report here the synthesis and conformational evaluation of the THBC-DKP-based peptidomimetic **1a** (Figure 2), for which molecular modeling allowed us to envisage a 13-membered hydrogen-bond-stabilized α-turn conformation.

## Results and Discussion

**Figure 3:**
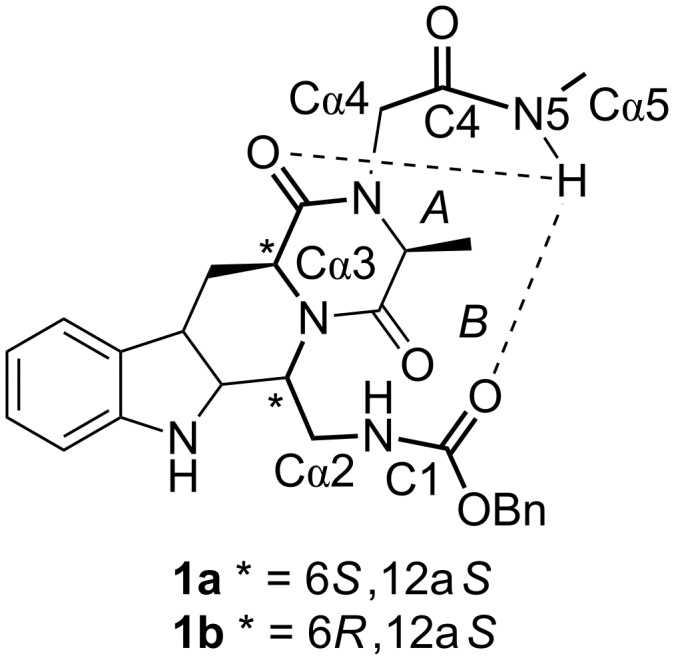
Geometric parameters for mimics **1a**,**b**.

In order to investigate the presence of a preferred conformation able to mimic an ordered protein secondary structure, a computer-aided conformational analysis was performed on **1a** (6*S*,12a*S*-configuration, IUPAC atom numbering as in Figure 2) and diastereoisomeric **1b** (6*R*, 12a*S*). Compounds **1a** and **1b** were submitted to an unconstrained Monte Carlo (MC) conformational search combined with Molecular Mechanics (MM) minimization (see Table 1 for results). As a main indication of a stable secondary structure, the presence of intramolecular hydrogen bonds was first evaluated. Two H-bonds have been identified, i.e., a 7-membered-ring H-bond (H-bond *A*, Figure 3) between the N5H hydrogen and the C3 carbonyl and a 13-membered-ring H-bond (H-bond *B*) between the N5H hydrogen and the C1 carbonyl, here represented by the Cbz carbonyl. To assess the presence of turn conformations we also measured the interatomic distance *d*α between the terminal Cα5 atom and the benzyl oxygen of the Cbz (which emulates the Cα1 atom), assuming a value *d*α < 7 Å as a probe of a generic reverse turn. Results are reported as the number of conformers that meet the geometric requirements.

**Table 1:** MC/EM conformational analysis for peptidomimetics **1a** and **1b**. The + symbol indicates the presence in the global minimum.

	Conf. within 6 kcal/mol	H-bond *A*	H-bond *B*	*d*α < 7 Å

**1a**	35	17	4 +	4 +
**1b**	33	22 +	1	1

**Figure 4:**
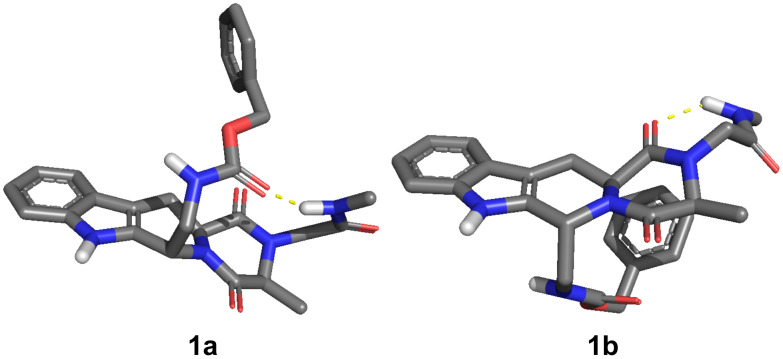
Perspective view of the low-energy conformers of **1a**,**b**. Hydrogen atoms are omitted for clarity.

The most frequently observed is the 5→3 H-bond (H-bond *A*), which is related to a classical γ-turn around these residues, and a 5→1 H-bond (H-bond *B*), which can be identified as an α-turn (this conformation is present in four conformers for compound **1a**). Inspection of the global minima showed the α-turn conformation only for compound **1a**. For this diastereoisomer the first four low-energy conformers all adopt the α-turn conformation (presence of the 5→1 H-bond and average *d*α = 5.1 Å): according to a Boltzmann distribution analysis, these conformers take into account 94.5% of all the conformers obtained by the MC/MM analysis. On the other hand, for compound **1b**, the lowest energy conformers all have the 5→3 H-bond (classical γ-turn), with the only α-turn conformation lying 5.9 kcal/mol above the global minimum. In Figure 4 the low-energy conformers of **1a** and **1b** are represented. These pictures highlight well the crucial role of the C6-configuration in favoring either an α-turn conformation (**1a**) or a γ-turn conformation (**1b**).

**Scheme 1:**
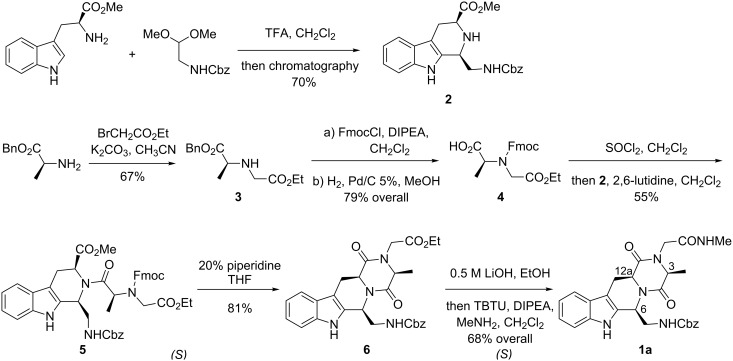
Synthesis of the THBC-DKP-based peptidomimetic **1a**.

Finally, having ascertained by means of further calculations the irrelevance of the C3 configuration on the expected secondary structure, we fixed it as 3*S*. Being interested in unusual reverse turns, we then pursued the synthesis of peptidomimetic **1a** (Scheme 1). Starting from L-tryptophan methyl ester and *N*-Cbz-aminoacetaldehyde dimethyl acetal [43], tetrahydro-β-carboline **2** was obtained in good yield and high diastereoselectivity (dr 70% from ^1^H NMR) by means of Pictet–Spengler reaction [44] and subsequent chromatographic separation.

The 6*S*,12a*S*-configuration of the prevailing diastereoisomer was easily ascertained by application of the protocol of Ungemach et al. [45] on the ^13^C NMR spectrum of compound **2** and, conclusively, by the observation of an intense NOE contact between H-6 and H-12a in the 2D NOESY spectrum. Alkylation of L-alanine benzyl ester with ethyl bromoacetate afforded amine **3** [46], on which *N*-Fmoc protection and subsequent carefully conducted hydrogenolysis of the benzyl ester were performed [47], to give acid **4** in acceptable overall yield.

Condensation of acid **4** with secondary amine **2** proved to be troublesome under a wide range of conditions, probably due to the severe steric hindrance of both the amine and acid coupling partners. At the end, acceptable yields could be obtained via formation of the chloride intermediate [48], by reaction of acid **4** with thionyl chloride, and subsequent coupling with **2** in CH_2_Cl_2_ and 2,6-lutidine to give **5**. The formation of the fused, tetracyclic THBC-DKP ring system was then easily achieved by removal of the *N*-Fmoc protecting group [49] and spontaneous lactamization to give the diketopiperazine ring of **6**. The obtained compound **6** represents a valuable peptidomimetic, whose potential is also related to the possibility of further derivatization with desired pharmacophoric groups, on both the terminal acid and amine functional groups, for the development of conformationally constrained tryptophan-containing peptide ligands.

To investigate the actual secondary structure of the THBC-DKP scaffold also in solution, compound **6** was converted into the *N*-methyl carboxyamide derivative **1a**, by a two-step procedure (0.5 M LiOH, 0 °C, then MeNH_2_, TBTU, DIPEA), which was carefully conducted in order to avoid the easy epimerization of the C3 and C12a stereocenters. Spin-system identification and assignment of individual resonances for peptidomimetic model **1a** was straightforward with ^1^H COSY. The study of the conformational behavior was conducted in CDCl_3_, to identify possible intramolecular hydrogen bonding.

The involvement of the NH amide protons in such bonding was first estimated from evaluation of their chemical shift value (δ) and of the temperature coefficients Δδ/Δ*T* (between 263 and 328 K). All data were measured at 2.0 mM concentration, that is, in the absence of any noticeable intermolecular aggregation. While both NHMe and NHCbz chemical-shift values are low (6.78 ppm for NHMe and 5.53 ppm for NHCbz), a significant difference could be appreciated between their temperature coefficients, ranging from 7.0 ppb K^−1^ (in absolute value) for the NHMe signal to 2.3 ppb K^−1^ (in absolute value) for the NHCbz signal. According to the literature [50], these data could be attributed to a situation of equilibrium between hydrogen-bonded and non-hydrogen-bonded states for the NHMe amide proton and a completely non-hydrogen-bonded state for the NHCbz amide proton. In addition, a supplementary indication of the different hydrogen-bonding state for the two NH protons was obtained from DMSO titration studies in CDCl_3_ [51], indicating that the chemical shift of the NHMe has a minor variation (0.24 ppm) upon addition of up to 30% of the competitive solvent DMSO, with respect to the chemical shift of NHCbz, i.e., varying by 0.82 ppm.

Taking into account the suggestions from the molecular modeling and these experimental results, for **1a** we can presume the presence in solution of conformers bearing a 13-membered intramolecular hydrogen bond involving the NHMe proton and the Cbz carbonyl group, as visualized in the perspective view of the low energy conformer of **1a**.

## Conclusion

In conclusion, we realized the synthesis of a new constrained THBC-DKP-based scaffold able to mimic an α-turn. It was designed with the aid of computational tools, which highlight the relative *cis* arrangement of the substituents on the THBC piperidine ring as being a crucial requirement in order to obtain the correct geometry for mimicry. Following these studies, the desired isomer **1a** of the THBC-DKP based peptidomimetic was synthesized. ^1^H NMR conformational studies confirmed the presence of the intramolecular thirteen-membered hydrogen bond that characterizes the α-turn conformation, even if a situation of equilibrium between hydrogen-bonded and non-hydrogen-bonded states can be observed. Nevertheless, this scaffold represents one of the rare examples of a designed constrained α-turn mimic. Its application to the synthesis of biologically valuable peptides embodying an α-turn core is currently under way.

## Experimental

**General information:** All commercial materials (Aldrich, Fluka) were used without further purification. All solvents were reagent grade or HPLC grade. All reactions were carried out under a nitrogen atmosphere unless otherwise noted. All reactions were monitored by thin-layer chromatography (TLC) on precoated silica gel 60 F_254_; spots were visualized with UV light or by treatment with 1% aqueous KMnO_4_ solution. Products were purified by flash chromatography on silica gel 60 (230–400 mesh). ^1^H NMR spectra and ^13^C NMR spectra were recorded on 300, 400 and 500 MHz spectrometers. Chemical shifts are reported in parts per million relative to the residual proton resonance of the solvent. ^13^C NMR spectra were recorded by using the APT pulse sequence. Multiplicities in ^1^H NMR are reported as follows: s = singlet, d = doublet, t = triplet, m = multiplet, br s = broad singlet. High-resolution MS spectra were recorded with a FT-ICR (Fourier Transform Ion Cyclotron Resonance) instrument, equipped with an ESI source, or a standard MS instrument, equipped with an EI source. Yields refer to chromatographically and spectroscopically pure compounds.

**(1*S*,3*S*)-Methyl 1-(benzyloxycarbonylaminomethyl)-2,3,4,9-tetrahydro-1*H*-pyrido[3,4-*b*]indole-3-carboxylate (2):** Under a nitrogen atmosphere, L-tryptophan methyl ester (755 mg, 3.46 mmol, 1.0 equiv) was dissolved in CH_2_Cl_2_ (30 mL, 0.12 M), then *N*-Cbz-aminoacetaldehyde dimethyl acetal (912 mg, 3.81 mmol, 1.1 equiv) was added. The solution was cooled to −30 °C, then TFA was added (1.97 mg, 1.3 mL, 17.3 mmol, 5 equiv). The mixture was kept for 2 h at this temperature then reacted overnight at room temperature. The solution was diluted with AcOEt and washed three times with a saturated solution of NaHCO_3_ (3 × 15 mL), dried over Na_2_SO_4_, filtered and evaporated under reduced pressure. The crude mixture was purified by chromatographic column (*n*-hexane/AcOEt 6:4), and 808 mg of (1*S*,3*S*)-**2** and 143 mg of the (1*R*,3*S*)-diastereoisomer were obtained (70% overall yield, 70% dr).

Characterization of **2**. Oil; [α]_D_ −44.4 (*c* 1, CHCl_3_); ^1^H NMR (CDCl_3_, 400 MHz) δ 8.96 (1H, br s), 7.55–7.10 (9H, m), 6.02 (1H, br, dd, NH-Cbz), 5.03 (2H, s), 4.40 (1H, br s), 3.90–3.60 (6H, m), 3.18 (1H, dd, *J* = 15.0, 1.6 Hz), 2.88 (1H, dd, *J* = 15.0, 11.2 Hz), 2.77 (1H, br s); ^13^C NMR (CDCl_3_, 100 MHz) δ 173.5, 157.3, 136.5, 136.3, 132.3, 128.5–127.8 (5C), 127.0, 122.0, 119.5, 118.0, 111.3, 109.2, 66.9, 56.3, 53.6, 52.3, 44.3, 25.5; IR (cm^−1^): 3018, 1709, 1514, 1362, 1267, 1228; HRMS–EI (*m*/*z*): [M^+^] calcd for C_22_H_23_N_3_O_4_, 393.1689; found, 393;1702.

**(*S*)-Benzyl 2-((2-ethoxy-2-oxoethyl)amino)propanoate (3):** Under a nitrogen atmosphere L-alanine-benzyl ester (500 mg, 2.79 mmol, 1 equiv) was dissolved in CH_3_CN (6 mL, 0.47 M), then K_2_CO_3_ (3.85 g, 27.90 mmol, 10 equiv) was added. Ethyl bromoacetate (466 mg, 2.79 mmol, 310 µL, 1 equiv) was added to the suspension, and the mixture was reacted overnight at room temperature. The solvent was evaporated under reduced pressure, and the crude material was dissolved in AcOEt (20 mL). The solution was extracted with an aqueous solution of H_3_PO_4_ 5% (3 × 25 mL), and the reunited aqueous layers were basified with Na_2_CO_3_ until pH 9 and extracted repeatedly with AcOEt. The reunited organic phases were dried with Na_2_SO_4_, filtered and evaporated under reduced pressure, to give compound **3** (503 mg, 67% yield), as an oil. [α]_D_ −17.11 (*c* 1, CHCl_3_); ^1^H NMR (CDCl_3_, 300 MHz) δ 7.4–7.28 (5H, m), 5.2–5.1 (2H, m), 4.15 (2H, q, *J* = 8.6 Hz), 3.53 (1H, q, *J* = 9.6 Hz), 3.49 (1H, d, *J* = 17.3 Hz), 3.39 (1H, d, *J* = 17.3 Hz), 2.37 (1H, br s), 1.38 (3H, d, *J* = 9.6 Hz), 1.25 (3H, t, *J* = 8.6 Hz); ^13^C NMR (CDCl_3_, 75.4 MHz) δ 174.3, 171.4, 135.6, 128.6–128.2 (5C), 66.7, 61.0, 55.9, 48.7, 18.6, 14.2; HRMS–EI (*m*/*z*): [M^+^] calcd for C_14_H_19_NO_4_, 265.1314; found, 265.1328.

**(*S*)-2-((9*H*-Fluoren-9-ylmethoxycarbonyl)(2-ethoxy-2-oxoethyl)amino)propanoic acid (4):** Under a nitrogen atmosphere, compound **3** (1.49 g, 5.62 mmol, 1 equiv) and DIPEA (871 mg, 6.74 mmol, 1.17 mL, 1.2 equiv) were dissolved in CH_2_Cl_2_ (40 mL, 0.14 M), then FmocCl (1.48 g, 5.73 mmol, 1.02 equiv) was added. The mixture was stirred at room temperature overnight. Then CH_2_Cl_2_ (10 mL) was added, the solution was washed with HCl 0.5 M (3 × 30 mL), and the organic layer was dried with Na_2_SO_4_, filtered and evaporated under reduced pressure. The crude was dissolved in MeOH (100 mL, 0.045 M), and then, under a nitrogen atmosphere, Pd/C 5% w/w (150 mg) was added. The resulting suspension was stirred for 3 h under a hydrogen atmosphere at room temperature. The reaction was accurately monitored by TLC in order to prevent hydrogenolysis of the Fmoc group. The suspension was filtered on a layer of celite, the solution was evaporated under reduced pressure, and the crude was dissolved in AcOEt and then extracted three times with a saturated solution of NaHCO_3_. The reunited aqueous phases were acidified with HCl (0.5 M) and extracted with AcOEt (3 × 35 mL), then dried with Na_2_SO_4_, filtered and evaporated under reduced pressure, to give pure **4** (1.76 g, 79% overall yield) as an oil. [α]_D_ −9.42 (*c* 1, MeOH); ^1^H NMR (DMSO, 300 MHz, 95 °C) δ 7.88 (2H, d, *J* = 7.5 Hz), 7.64 (2H, d, *J* = 7.4 Hz), 7.43 (2H, t, *J* = 7.3 Hz), 7.34 (2H, t, *J* = 7.3 Hz), 4.58 (1H, q, *J* = 7.3 Hz), 4.38 (2H, d, *J* = 6.3 Hz), 4.26 (1H, t, *J* = 6.3 Hz), 4.12 (2H, q, *J* = 7.1 Hz), 3.98 (2H, AB system, *J* = 18.0 Hz), 1.37 (3H, d, *J* = 7.3 Hz), 1.21 (3H, t, *J* = 7.1 Hz); ^13^C NMR (CDCl_3_, 75.4 MHz, mixture of two rotamers) δ 175.4 and 174.5, 171.8 and 170.7, 155.7 and 155.6, 143.7, 143.6, 141.2 (2C), 127.7 (2C), 127.1 (2C), 124.9 (2C), 120.0 (2C), 68.6 and 68.2, 62.2 and 61.8, 55.7 and 55.5, 47.6 and 47.1, 47.0, 15.5 and 14.9, 14.1; IR (cm^−1^): 2954, 1750, 1709, 1467, 1451, 1325, 1204; HRMS–EI (*m*/*z*): [M^+^] calcd for C_22_H_23_NO_6_, 397.1525; found, 397.1541.

**(1*S*,3*S*)-Methyl 2-((*S*)-2-((9*H*-fluoren-9-ylmethoxycarbonyl)(2-ethoxy-2-oxoethyl)amino)propanoyl)-1-(benzyloxycarbonylaminomethyl)-2,3,4,9-tetrahydro-1*H*-pyrido[3,4-*b*]indole-3-carboxylate (5):** Under a nitrogen atmosphere, compound **4** (302 mg, 0.76 mmol, 1 equiv) was dissolved in CH_2_Cl_2_ (4 mL, 0.19 M), the solution was cooled to 0 °C, and then SOCl_2_ (904 mg, 7.60 mmol, 550 µL, 10 equiv) was added. The mixture was stirred at room temperature for 5 h then evaporated under reduced pressure. The obtained crude material was dissolved in CH_2_Cl_2_ (3 mL) and slowly added to a solution of **2** (299 mg, 0.76 mmol, 1 equiv) and 2,6-lutidine (110 mg, 1.03 mmol, 120 μL, 1.35 equiv) in CH_2_Cl_2_ (5 mL), cooled at 0 °C under nitrogen atmosphere. The mixture was stirred at room temperature overnight, then CH_2_Cl_2_ (10 mL) was added, the organic layer was washed with HCl (0.5 M, 5 mL), saturated aq NaHCO_3_ (5 mL) and brine (5 mL). The organic phase was dried with Na_2_SO_4_, filtered and evaporated under reduced pressure. The crude material was purified by column chromatography (*n*-hexane/AcOEt 6:4), affording **5** (321 mg, 55% yield) as an oil. [α]_D_ +17.87 (*c* 1 CHCl_3_), ^1^H NMR (CDCl_3_, 300 MHz, major rotamer) δ 9.87 (1H, br s), 7.88–7.00 (17H, m), 6.07 (1H, br s), 5.45 (1H, br q), 5.32 (1H, br s), 5.23 (2H, br s), 4.71–4.32 (2H, m), 4.20–4.10 (5H, m), 4.08–3.74 (3H, m), 3.74–3.08 (5H, m), 1.48 (3H, d, *J* = 7.5 Hz), 1.25 (3H, d, *J* = 7.3 Hz); ^13^C NMR (CDCl_3_, 100 MHz, major rotamer) δ 171.1, 169.3, 169.1, 155.9, 154.2, 143.9, 141.2, 136.4, 130.6, 128.5–111.5 (22C), 68.8, 67.1, 61.4, 56.3, 54.8, 52.1, 51.0, 47.1, 47.0, 46.6, 24.4, 15.3, 14.2; HRMS–EI (*m*/*z*): [M^+^] calcd for C_44_H_44_N_4_O_9_, 772.3108; found, 772.3095.

**Ethyl 2-((3*S*,6*S*,12a*S*)-6-(benzyloxycarbonylaminomethyl)-3-methyl-1,4-dioxo-3,4,12,12a-tetrahydropyrazino[1',2':1,6]pyrido[3,4-*b*]indol-2(1*H*,6*H*,7*H*)-yl)acetate (6):** Compound **5** (300 mg, 0.39 mmol) was dissolved in THF (13 mL, 0.03 M), and then piperidine was added (2.6 mL). The solution was reacted overnight, then HCl (0.5 M) was added, and the mixture was extracted with AcOEt (3 × 30 mL). The reunited organic layers were dried with Na_2_SO_4_, filtered and evaporated under reduced pressure. The crude material was purified by column chromatography (AcOEt/*n*-hexane, 4:6 to 6:4), to give **6** (165 mg, 81% yield) as an oil. [α]_D_ −59.5 (*c* 1, CHCl_3_); ^1^H NMR (CDCl_3_, 400 MHz, major rotamer) δ 9.27 (1H, br s), 7.55–7.10 (9H, m), 5.91 (1H, br s), 5.15–5.09 (3H, m), 4.50 (1H, d, *J* = 17.2 Hz), 4.26 (4H, m), 4.05 (1H, m), 3.97 (1H, d, *J* = 17.5 Hz), 3.68 (1H, m), 3.57 (1H, br d, *J* = 15.6 Hz), 3.07 (1H, dd, *J* = 15.6, 11.8 Hz), 1.58 (3H, d, *J* = 12.8 Hz), 1.31 (3H, t, *J* = 7.2 Hz); ^13^C NMR (CDCl_3_, 75.4 MHz) δ 168.9, 168.5, 167.3, 157.6, 136.5, 136.3, 130.7, 128.6, 128.2, 128.0, 126.2, 122.5, 119.9, 119.8, 118.3, 111.7, 107.7, 67.1, 61.8, 57.6, 57.2, 56.3, 45.0, 44.7, 31.6, 25.2, 17.9, 14.2; IR (cm^−1^): 3016, 1742, 1667, 1506, 1455, 1326, 1222. HRMS–EI (*m*/*z*): [M^+^] calcd for C_28_H_30_N_4_O_6_, 518.2165; found, 518.2178.

**Benzyl (((3*S*,6*S*,12a*S*)-3-methyl-2-(2-(methylamino)-2-oxoethyl)-1,4-dioxo-1,2,3,4,6,7,12,12a-octahydropyrazino[1',2':1,6]pyrido[3,4-*b*]indol-6-yl)methyl)carbamate (1a):** Compound **6** (165 mg, 0.32 mmol, 518 g/mol) was dissolved in EtOH (3 mL). Aqueous LiOH (0.5 M, 2.54 mmol, 4.6 mL) was added and the reaction was stirred at 0 °C for 1 h. After then, the solution was acidified with HCl (1 N, 8 mL), extracted with AcOEt (3 × 20 mL), dried, filtered and concentrated. The crude material was suspended in CH_2_Cl_2_ (10 mL) under N_2_, DIPEA (208 µL, 1.20 mmol, 129 g/mol) and TBTU (384 mg, 1.20 mmol, 321 g/mol) were added. After stirring for 30 min, MeNH_2_ (2.0 M THF solution, 1.20 mmol, 601 µL) was added. The reaction was stirred for 24 h under N_2_, and then it was poured into water (20 mL) and extracted with AcOEt (3 × 20 mL), dried, filtered and concentrated. Purification by flash chromatography (AcOEt) afforded product **1a** (110 mg, 68% overall yield) as an oil. [α]_D_ −98.3 (*c* 0.75, MeOH); ^1^H NMR (500 MHz, CD_3_CN, major rotamer) δ 9.41 (1H, br s), 7.55 (1H, d, *J* = 7.6 Hz), 7.38 (1H, d, *J* = 7.8 Hz), 7.34–7.23 (5H, m), 7.13 (1H, ddd, *J* = 7.8, 7.6 and 0.9 Hz), 7.06 (1H, dt, *J* = 7.6 and 0.9 Hz), 6.95 (1H, br q, *J* = 4.7 Hz), 5.83 (1H, br q, *J* = 8.2 and 5.1 Hz), 5.43 (1H, br t, *J* = 3.4 Hz), 4.90 (1H, d, *J* = 12.3 Hz), 4.87 (1H, d, *J* = 12.3 Hz), 4.26 (1H, d, *J* = 17.0 Hz), 4.16 (1H, q, *J* = 7.0 Hz), 4.14 (1H, dd, *J* = 11.4 and 5.5 Hz), 3.98 (1H, ddd, *J* = 14.2, 8.2 and 3.8 Hz), 3.94 (1H, d, *J* = 17.0 Hz), 3.43 (1H, dd, *J* = 15.6 and 5.5 Hz), 3.26 (1H, ddd, *J* = 14.2, 5.1 and 3.2 Hz), 3.00 (1H, dd, *J* = 15.6 and 11.4 Hz), 2.69 (3H, d, *J* = 4.7 Hz), 1.48 (3H, d, *J* = 7.0 Hz); ^13^C NMR (125.8 MHz, CD_3_CN) δ 170.4, 170.0, 169.8, 157.8, 137.5, 135.2, 132.0, 129.0 (2C), 128.5, 128.3 (2C), 126.9, 122.2, 119.8, 118.7, 112.0, 108.1, 66.7, 55.4, 55.3, 53.5, 46.7, 44.7, 22.7, 25.9, 14.5; HRMS–EI (*m*/*z*): [M^+^] calcd for C_27_H_29_N_5_O_5_, 503.2169; found, 503.2186.

### Computational details

An unconstrained Monte Carlo (MC) conformational search combined with Molecular Mechanics (MM) minimization was performed by using the software Spartan’08 [52]. Images were elaborated with the software PyMol [53]. For each compound 972 conformers were generated, and after removal of duplicated minima, only conformations within 6 kcal/mol of the global minimum were considered. Thirty-five and thirty-three conformers were kept for compounds **1a** and **1b**, respectively. Inspection of the virtual torsion angles of the amide backbone revealed the preference for an inverse γ-turn of the low-energy conformers of **1b** (φ = −80.1° and ψ = 79.6° on average, based on the conformers accounting 93% of all the conformers from a Boltzmann distribution analysis). For compound **1a** the averages of the torsion angles were φ (C_α2_) = −81.5°, ψ (C_α2_) = 64.5°, φ (C_α3_) = 146.3°, ψ (C_α3_) = 36.9°, φ (C_α4_) = 120.5°, and ψ (C_α4_) = −29.3°. According to the classification of α-turns in protein structures [32], compound **1a** is an α-turn mimetic with a B-α_L_-X designation.
